# Extracellular vesicle-mediated immune remodeling in cutaneous wound repair

**DOI:** 10.3389/fimmu.2026.1938968

**Published:** 2026-10-06

**Authors:** Gun Woo Lee, Wook-Tae Park, Min-Soo Seo, Sangbum Park

**Affiliations:** 1 Department of Orthopedic Surgery, Ulsan Medical Center, Ulsan, Republic of Korea; 2 Cellexobio, Ltd., Daegu, Republic of Korea; 3 Institute for Quantitative Health Science and Engineering (IQ), Michigan State University, East Lansing, MI, United States; 4 Department of Orthopedic Surgery, Yeungnam University College of Medicine, Daegu, Republic of Korea; 5 Laboratory of Veterinary Tissue Engineering, College of Veterinary Medicine, Kyungpook National University, Daegu,Republic of Korea; 6 Department of Medicine, College of Human Medicine, Michigan State University, East Lansing, MI, United States; 7 Department of Pharmacology and Toxicology, College of Human Medicine, Michigan State University, East Lansing, MI, United States

**Keywords:** extracellular vesicles, immune remodeling, inflammation, skin regeneration, wound healing

## Abstract

Extracellular vesicles (EVs) are emerging cell-free therapeutic platforms for cutaneous wound repair that regulate diverse cellular processes, including intercellular communication, inflammation, angiogenesis, extracellular matrix remodeling, and tissue repair. Recent advances in parental cell preconditioning, EV engineering, smart EV design, and biomaterial-assisted delivery have further enhanced the therapeutic potential of EV-based approaches. These advances have stimulated growing interest in applying EVs to skin wound repair, where EVs act on resident skin cells and immune cells to promote regenerative healing. In particular, macrophage reprogramming is well characterized, whereas EV-mediated regulation of other immune-cell populations remains less clearly defined. Standardized EV production, reliable potency assays, and dynamic *in vivo* approaches will be essential for defining how EVs coordinate regenerative and immune-regulatory processes. This review discusses EV-mediated cutaneous wound repair with emphasis on temporal and cell-specific immune remodeling, regenerative mechanisms, and translational challenges.

## 1 Introduction

Skin wound repair is a highly organized process involving overlapping phases of hemostasis, inflammation, proliferation, and remodeling, during which distinct immune-cell populations act in a temporally coordinated manner. Neutrophils and inflammatory monocyte/macrophages dominate the early response, after which macrophage phenotypic transitions drive inflammatory resolution and tissue repair, while adaptive immune populations contribute to later-stage regulation (1). Dysregulation of this coordinated sequence can lead to delayed healing, chronic wounds, or excessive fibrosis (2).

Extracellular vesicles (EVs) are membrane-bound particles released by cells that carry diverse molecular cargo and mediate intercellular communication (3). In cutaneous wounds, EVs can regulate both resident skin cells and immune cells, thereby influencing re-epithelialization, angiogenesis, ECM remodeling, and inflammation. EV cargo and biological activity are shaped by the cellular source and local microenvironment, including inflammatory, hypoxic, and mechanical conditions, suggesting that EV-mediated signaling may change dynamically during wound repair (4, 5).

A major conceptual advance in regenerative medicine is the paradigm shift from direct stem cell replacement to paracrine regenerative signaling. Accumulating evidence indicates that the therapeutic effects of mesenchymal stem cells (MSCs) are mediated primarily through their secretome, particularly EV-associated bioactive cargo, rather than durable engraftment or direct differentiation into tissue-specific cells. This recognition has generated significant interest in MSC-derived EVs as cell-free therapeutic platforms that reproduce many benefits of stem cell therapy while avoiding major limitations of whole-cell transplantation (6).

This review therefore focuses on EV-mediated immune remodeling during cutaneous wound repair, with emphasis on temporal and cell-specific responses. We also discuss EV design, delivery, and regenerative outcomes primarily in relation to their effects on the wound immune microenvironment.

## 2 EV properties and delivery strategies shaping immune responses

The immunomodulatory activity of EVs is shaped by three interconnected factors: the cellular source and preconditioning of parental cells, EV cargo composition and engineering, and the mode of delivery. These factors influence EV bioactivity, tissue retention, and immune-cell exposure within the wound microenvironment.

Studies report that the biological activity of EVs depends on their cellular origin and microenvironmental conditioning. MSC secretomes derived from bone marrow, adipose tissue, and umbilical cord display conserved and tissue-specific bioactive signatures that differentially regulate inflammation, angiogenesis, fibrosis, oxidative stress, and tissue repair. Umbilical cord-derived MSC-EVs often show potent anti-inflammatory and regenerative effects, whereas adipose-derived MSC-EVs tend to exhibit enhanced angiogenic and ECM-remodeling activity. This source-dependent heterogeneity highlights the need to optimize donor selection, culture conditions, hypoxic priming, inflammatory preconditioning, and bioreactor systems to improve therapeutic efficacy and reproducibility (4). Beyond cellular origin, biomechanical cues can influence EV biogenesis, composition, and function. Mechanical forces, including fluid shear stress, cyclic stretch, tensile strain, and ECM stiffness, can regulate EV secretion and cargo composition (5).

Building on these insights, engineering strategies have been developed to further optimize EV therapeutic performance. These approaches are designed to address limitations of native EVs, including rapid clearance, low tissue specificity, heterogeneous cargo composition, and limited therapeutic potency. Engineered EVs can be modified through genetic engineering, chemical conjugation, physical modification, membrane hybridization, and cargo-loading technologies to enhance targeting and therapeutic efficacy. Targeting peptides, antibodies, aptamers, and imaging probes may be incorporated into EV membranes to optimize biodistribution and enable precision therapy (7). In skin regeneration, engineered EVs enriched with pro-angiogenic miRNAs, anti-inflammatory molecules, or antioxidant cargos may further enhance wound repair compared with naïve EVs. More recently, smart EVs have been engineered with stimuli-responsive properties that enable controlled activation in pathological microenvironments, including oxidative stress, acidic pH, inflammatory cytokines, or reactive oxygen species (ROS). Such systems may provide spatially and temporally controlled cargo delivery (8). In chronic or inflamed wounds, these systems may help restrict therapeutic activity to pathological microenvironments while limiting off-target effects.

Biomaterial-assisted EV delivery systems have also emerged as key strategies to enhance therapeutic retention, stability, and sustained release. Hydrogels, nanofiber scaffolds, collagen matrices, microneedles, and three-dimensional printed biomaterials serve as localized delivery platforms that protect EVs from degradation and prolong bioactivity in injured tissues (9). By improving local EV availability and release kinetics, these systems may prolong EV exposure to resident and immune cells within the wound.

## 3 Regenerative effects of EVs in cutaneous wound repair

MSC-derived EVs regulate wound repair through paracrine transfer of proteins and regulatory RNAs to recipient skin cells (10). EV-associated miRNAs contribute to fibroblast and keratinocyte responses; for example, miR-21 promotes fibroblast proliferation through PTEN inhibition and PI3K/Akt activation, whereas miR-146a suppresses NF-κB-dependent inflammatory signaling (11). Several signaling pathways converge on these effects, including PI3K/Akt, Wnt/β-catenin, and Nrf2-mediated antioxidant signaling, which support fibroblast survival, keratinocyte proliferation, re-epithelialization, and protection from oxidative stress during repair (11, 12). Together, these effects help restore epithelial integrity while supporting stromal-cell survival and function within the wound bed.

EVs also promote angiogenesis by supporting endothelial migration, proliferation, and tube formation. Newly formed vasculature restores oxygen and nutrient delivery while facilitating immune-cell trafficking within the wound. This pro-angiogenic activity is influenced by EV source and conditioning, which can alter the abundance and activity of angiogenic cargo delivered to endothelial cells. Inflammatory preconditioning can enhance this response: EVs derived from TNF-α- and IL-1β-preconditioned human umbilical cord MSCs accelerate wound closure and CD31-positive angiogenesis, with miR-215-5p and WNK1/p-Smad3/VEGF-A signaling identified as key mediators (13). In addition, the composition of endogenous wound-derived EVs changes with wound age and genetic background, suggesting that EV-mediated signaling may vary across different stages and conditions of repair (14).

EV treatment can also influence fibroblast differentiation, collagen deposition, and scar formation by modulating ECM remodeling pathways. In particular, regulation of TGF-β/Smad signaling and myofibroblast activation may help limit excessive fibrosis while preserving tissue repair. Because persistent myofibroblast activation and abnormal collagen accumulation contribute to hypertrophic scarring and fibrotic repair, EV-mediated modulation of these processes may help restore a more balanced remodeling response. EV treatment has also been associated with reduced α-smooth muscle actin expression and improved collagen organization in experimental wound and scar models (12). These regenerative outcomes are closely linked to changes in the wound immune microenvironment, which are discussed in the following section.

## 4 Temporal and cell-specific immune remodeling by EVs during wound repair

The regenerative outcomes of EV treatment are closely linked to the timely regulation and resolution of inflammation, making immune remodeling a central mechanism of EV-mediated skin regeneration (1). Yet this process is not driven by a single immune cell type acting alone. Several immune populations contribute at different times and locations within the wound (12). Because EV cargos can alter immune cell activation, cytokine production, metabolic state, and migratory behavior, these cell type-specific responses are central to understanding EV-mediated skin repair (**Figure 1**). Importantly, the strength of evidence differs substantially among immune-cell populations. Direct mechanistic evidence is strongest for macrophages and is emerging for neutrophils and dendritic cells, whereas evidence for T cells is largely extrapolated from non-wound models and direct evidence for Langerhans cells is currently lacking (**Table 1**).

**Figure 1:**
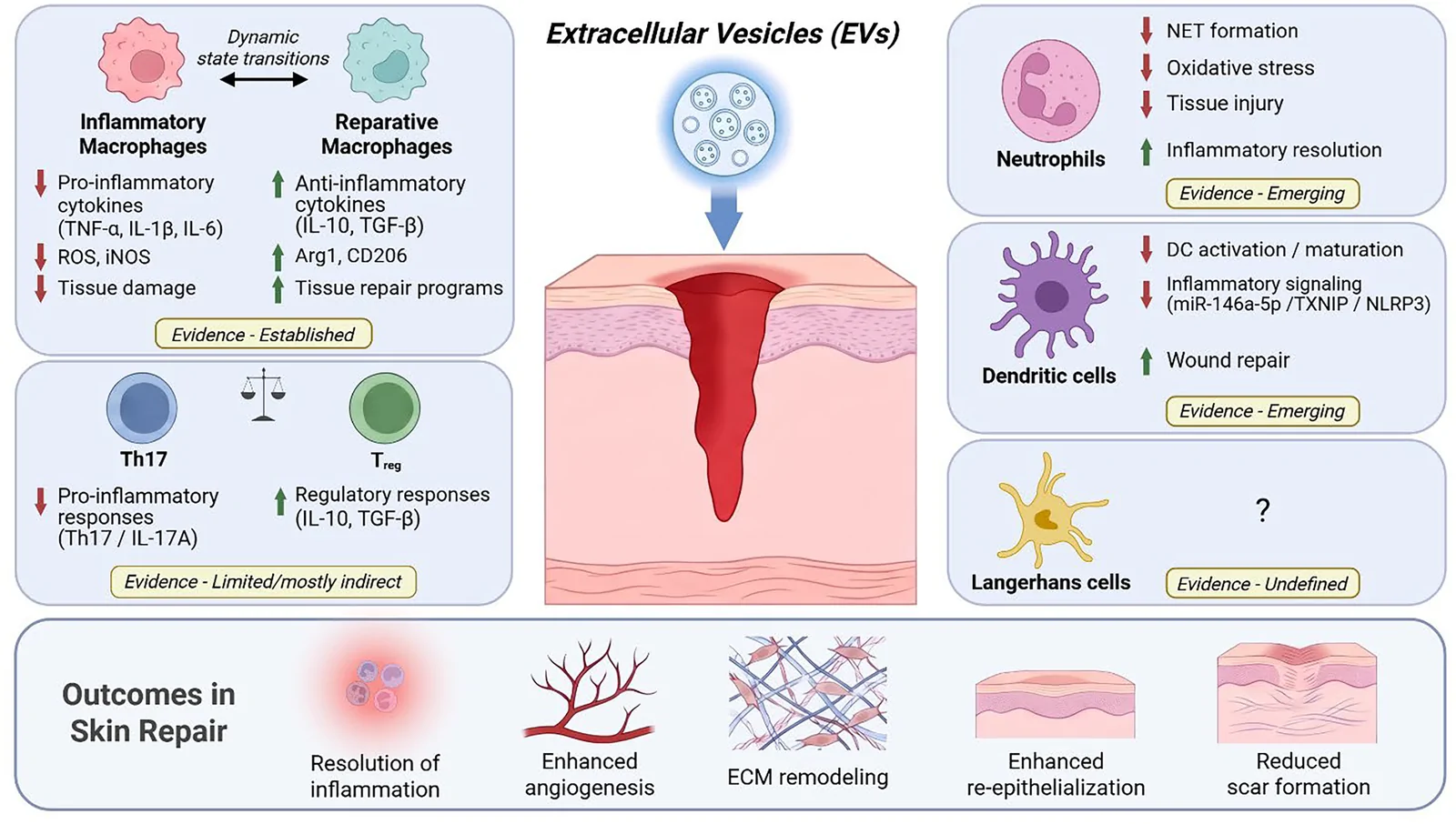
EV-mediated immune remodeling during cutaneous wound repair. EVs reshape the wound immune microenvironment by coordinating immune-cell activation, inflammatory resolution, and crosstalk with resident skin cells. These effects support inflammatory resolution and regenerative tissue remodeling. Created in BioRender. Park, S. (2026) https://BioRender.com/wuqj2yp.

**Table 1:** Representative evidence for EV-mediated immune remodeling in cutaneous wound repair.

Study	EV source/model	Immune target	Proposed mechanism	Immune outcome	Regenerative outcome	Evidence level	Key limitation
Kim H et al., 2019 (16)	Reparative macrophage-derived EVs; murine cutaneous wound	Macrophages	EV-mediated macrophage phenotypic transition	↓ Pro-inflammatory markers, ↑ reparative markers	Accelerated wound closure	Direct mechanistic	Limited macrophage-state resolution
Zhou X et al., 2020 (17)	Keratinocyte-derived EVs; murine cutaneous wound	Macrophages	Epithelial-to-macrophage EV transfer	Promoted macrophage-mediated inflammatory resolution	No significant change in wound closure or re-epithelialization	Direct mechanistic	Macrophage-focused analysis
Chen C et al., 2025 (18)	Fibroblast-derived EVs; cutaneous and diabetic wound models	Macrophages	Fibroblast-to-macrophage EV signaling; regulation of macrophage state transitions	Normalized macrophage activation; reduced aberrant inflammation	Improved diabetic wound healing	Direct mechanistic	Macrophage-focused mechanism
Li W et al., 2025 (19)	MSC-sEVs + GelMA microspheres; diabetic wound	Macrophages	p38 MAPK inhibition	↑ Reparative macrophage features	Improved diabetic wound healing	Direct mechanistic	Macrophage-focused mechanism
Kwak G et al., 2022 (20)	Reparative macrophage-derived EVs + PEG hydrogel; cutaneous wound	Macrophages	Sustained local EV release	Shift toward reparative macrophage states *in vivo*	Improved wound healing	Direct mechanistic	Limited immune-cell scope
Chu Z et al., 2023 (22)	Hypoxia-preconditioned MSC-sEVs; diabetic wound	Neutrophils	miR-17-5p-mediated inhibition of the TLR4/ROS/MAPK pathway; NET suppression	↓ NET formation	Improved diabetic wound healing	Direct mechanistic	NET-focused readouts
Lu W et al., 2024 (23)	MSC-derived EVs; diabetic wound	Neutrophils	Mitochondrial transfer to neutrophils; inhibition of NET-induced endothelial ferroptosis	↓ NET formation	Improved diabetic wound healing	Direct mechanistic	NET–endothelial axis focus
Rui S et al., 2024 (24)	PRP-derived EVs carrying miR-26b-5p; diabetic wound	Neutrophils	miR-26b-5p targets MMP-8	↓ NET formation	Improved diabetic wound healing	Direct mechanistic	Single miRNA/NET axis
Choi W et al., 2026 (25)	Endogenous wound-derived sEVs; murine cutaneous wound	Neutrophils	Rab27A-dependent sEV release	Delayed early neutrophil recruitment after impaired sEV release	Not directly assessed	Direct mechanistic	EV source/cargo-specific effects remain incompletely defined
Li J et al., 2025 (26)	TNF-α-primed ADSC EVs; diabetic (T2DM) wound	Dendritic cells	miR-146a-5p/TXNIP/NLRP3 axis	↓ Pathological DC activation	Improved diabetic wound healing	Direct mechanistic	Single DC pathway
Jung S et al., 2023 (30)	MSC-sEVs; non-wound inflammatory model	T cells (Th17)	Post-translational destabilization of RORγt	↓ Th17 responses	Not assessed in a wound model	Indirect/extrapolated	Non-wound model
Zhang B et al., 2018 (31)	MSC-derived EVs; general immunology (not a cutaneous wound model)	T cells (Treg)	APC-mediated pathway	↑ Regulatory T-cell production	Not assessed in a wound model	Indirect/extrapolated	Non-wound model
Babatunde KA et al., 2025 (32)	Macrophage-derived EVs; human microphysiological wound model	Neutrophils	EV-associated IL-8-mediated neutrophil reverse migration	↑ Neutrophil reverse migration	Not directly assessed	Direct mechanistic	*In vitro* microphysiological model
Park DJ et al., 2024 (33)	Wound-derived EVs; murine cutaneous and diabetic wound models, lineage mapping	Macrophages (EV source)	Macrophage-derived EV release and fibroblast uptake	Macrophages identified as a major EV source	Not directly assessed	Direct observational/mapping	No causal immune perturbation

Evidence categories: 1) Direct mechanistic, evidence from wound or wound-relevant models with perturbation or pathway-level analysis linking EV activity to a defined immune response; 2) Direct observational/mapping, direct evidence of EV source, localization, transfer, or uptake without causal perturbation; 3) Indirect/extrapolated, evidence derived mainly from non-wound models or broader immunological contexts.

EV, extracellular vesicle; MSC, mesenchymal stem cell; ADSC, adipose-derived mesenchymal stem cell; sEV, small extracellular vesicle; GelMA, gelatin methacryloyl; PEG, polyethylene glycol; PRP, platelet-rich plasma; NET, neutrophil extracellular trap; DC, dendritic cell; T2DM, type 2 diabetes mellitus; APC, antigen-presenting cell; Treg, regulatory T cell; Th17, T helper 17 cell.

Studies are organized by EV source/model, targeted immune-cell population, proposed mechanism, immune and regenerative outcomes, evidence level, and key limitation.

Macrophages are the best-characterized immune targets of EV therapy in cutaneous wound healing. During normal repair, inflammatory macrophages accumulate early after injury and contribute to debris clearance, antimicrobial defense, and production of inflammatory cytokines. As healing progresses, macrophages gradually acquire pro-resolving and tissue-reparative features that support angiogenesis, fibroblast function, collagen remodeling, and epithelial repair (15). Several studies indicate that EVs can accelerate this phenotypic transition. EVs derived from reparative macrophages can directly promote the transition of inflammatory macrophages toward a reparative phenotype, increasing reparative markers while reducing inflammatory features (16). Similarly, keratinocyte-derived EVs released from the wound edge have been shown to be selectively taken up by wound macrophages and to promote inflammatory resolution, suggesting that epithelial cells may use EVs as endogenous signals to instruct immune remodeling during tissue repair (17). Fibroblast-derived EVs have also been shown to regulate macrophage activation dynamics during skin wound healing, further indicating that resident stromal cells can shape macrophage behavior through EV-mediated communication (18). These findings support the concept that EV therapy may work, at least in part, by restoring or amplifying endogenous communication between resident skin cells and immune cells.

Sustained local EV exposure can further strengthen macrophage-directed immunoregulation. EV-based approaches can reduce pro-inflammatory signaling and promote macrophage transitions toward tissue-reparative states, as reflected by decreased iNOS, TNF-α, IL-1β, and IL-6, together with increased Arg1, CD206, and IL-10 in different experimental systems (16, 19). Sustained delivery of reparative macrophage-derived EVs through hydrolytically degradable polyethylene glycol (PEG) hydrogels prolonged local EV retention and supported a shift toward reparative macrophage states in cutaneous wounds (20). More recently, MSC-derived small EVs delivered through gelatin methacryloyl (GelMA) microspheres promoted the shift toward reparative macrophage features in diabetic wounds, partly through inhibition of p38 MAPK signaling (19).

Neutrophils are among the earliest immune cells recruited after skin injury and are essential for antimicrobial defense, but excessive or persistent neutrophil activity can amplify oxidative stress, protease activity, NET formation, and tissue damage (21). Recent studies indicate that MSC-derived EVs, hypoxia-preconditioned MSC-sEVs, and platelet-rich plasma (PRP)-derived EVs can suppress excessive NET formation and improve diabetic wound repair through mechanisms involving mitochondrial transfer, miR-17-5p, or miR-26b-5p (22–24). Beyond NET suppression, endogenous wound-derived sEVs also regulate neutrophil recruitment. Disruption of Rab27A-dependent sEV release reduced neutrophil and macrophage-derived sEVs and delayed early neutrophil recruitment in cutaneous wounds, indicating that endogenous EV signaling contributes to the timing of the acute inflammatory response (25). Thus, direct wound evidence for EV-mediated neutrophil regulation is emerging, encompassing both the control of early neutrophil recruitment and the suppression of excessive NET formation, although effects on neutrophil survival, phenotypic heterogeneity, and clearance remain poorly understood.

Antigen-presenting cells are emerging targets of EV-based skin repair. Dendritic cells and Langerhans cells occupy a central position between epithelial injury and adaptive immune activation. Evidence for antigen-presenting cells is more limited than for macrophages. In a diabetic wound model, TNF-α-primed adipose-derived MSC EVs suppressed pathological dendritic-cell activation through the miR-146a-5p/TXNIP/NLRP3 axis and accelerated healing (26), providing direct evidence for EV-mediated dendritic cell (DC) regulation in wounds. By contrast, proposed effects of EVs on antigen presentation and DC–T-cell interactions are derived mainly from broader EV immunology rather than cutaneous wound models (27). Direct evidence that EVs regulate Langerhans cell activation, migration, or repopulation during cutaneous wound repair is currently lacking.

Adaptive immune responses may also be influenced by EV treatment during skin regeneration. Chronic wounds can display sustained inflammatory cytokine production and altered T cell responses, including Th17- or IL-17A-associated inflammation in certain contexts (28, 29). However, direct evidence that therapeutic EVs regulate T cells in cutaneous wound models remains limited. MSC-EVs and other immunomodulatory EVs have been reported to suppress excessive effector T cell responses and favor regulatory or pro-resolving immune states, but these mechanisms are supported primarily by non-wound immunological models (30, 31). Thus, future studies should distinguish whether EVs directly regulate T cells or act indirectly through other cell types, such as macrophages, dendritic cells, keratinocytes, and stromal cells.

These immune-cell responses are not independent but are temporally and functionally interconnected. Neutrophils dominate the early inflammatory phase, while subsequent macrophage transitions contribute to inflammatory resolution and tissue repair (1). Recent evidence further indicates that macrophage-derived EVs can directly regulate neutrophil behavior: in a human microphysiological wound model, macrophage-derived EVs promoted neutrophil reverse migration through EV-associated IL-8, providing direct evidence for EV-mediated neutrophil–macrophage crosstalk during inflammatory resolution (32). Antigen-presenting cells can connect local tissue injury to adaptive T-cell responses, and T cells may further shape macrophage and stromal-cell activity during later stages of repair (27, 29). EVs may therefore influence wound healing not only by acting on individual immune populations but also by modifying communication among innate, adaptive, epithelial, and stromal compartments. Defining how these interactions change over time will be important for determining when and where EV therapy exerts its strongest immunomodulatory effects.

Overall, EV-mediated immune regulation in skin repair is a dynamic, cell-type-specific process rather than a uniform anti-inflammatory response. A major unresolved issue is that many EV wound-healing studies rely on endpoint histology, bulk cytokine measurements, or static marker analysis. While these approaches establish important associations, they do not fully capture the dynamic behavior of immune cells *in vivo*. Recent studies have begun to apply single-cell profiling and cell type-specific EV lineage-mapping approaches to trace EV source and uptake in the wound bed (33). Therefore, future EV studies should more broadly incorporate intravital imaging, spatial transcriptomics, single-cell profiling, lineage mapping, and quantitative immune-cell tracking to define how EV treatment changes immune cell recruitment, motility, cell–cell crosstalk, and residence within regenerating tissue.

## 5 Conclusion and future perspectives

EVs represent promising cell-free therapeutic platforms for regenerative medicine because of their ability to regulate intercellular communication, immune responses, angiogenesis, extracellular matrix remodeling, and tissue repair. In cutaneous wound repair, EVs can act on multiple cellular compartments, including keratinocytes, fibroblasts, endothelial cells, and immune cells, thereby supporting wound closure, vascularization, matrix remodeling, and scar modulation. Emerging strategies such as parental cell preconditioning, EV engineering, smart EV design, and biomaterial-assisted delivery further expand the therapeutic potential of EV-based approaches.

Despite these advances, several important challenges remain, including EV heterogeneity, dosing, delivery, potency evaluation, long-term safety, and regulatory classification. Standardized methods for EV isolation, purification, characterization, storage, dose normalization, and potency assessment are still needed to improve reproducibility across studies (3). Methodological heterogeneity in EV preparation may contribute to variability in the reported immune effects across studies, making it difficult to distinguish biological variation from methodological effects. Scalable GMP-compliant production also remains a major translational challenge. Beyond these general translational barriers, a central gap specific to cutaneous wound repair is the incomplete and cell-type-dependent understanding of EV-mediated immune regulation. Macrophage-targeted EV effects are relatively well characterized, whereas the effects of EV treatment on neutrophils, dendritic cells, Langerhans cells, and T cells remain less clearly defined. Future studies should therefore determine how EV therapies regulate immune-cell recruitment, migration, activation, resolution, and tissue residence *in vivo*. Combining standardized EV production with mechanistic approaches such as intravital imaging, single-cell analysis, spatial profiling, lineage mapping, quantitative immune-cell tracking, and biomaterial-controlled delivery will be essential for translating EV-based therapeutics into clinically applicable strategies for wound healing and scar prevention. Together, these advances suggest that the therapeutic value of EVs in skin repair depends not only on their direct regenerative effects on resident skin cells but also on their ability to reshape the immune microenvironment.
